# Preliminary studies on apparent mendelian psychotic disorders in consanguineous families

**DOI:** 10.1186/s12888-022-04304-4

**Published:** 2022-11-16

**Authors:** Ambreen Kanwal, Sohail A. Sheikh, Amina Iftikhar, Sadaf Naz, José V. Pardo

**Affiliations:** 1 School of Biological Sciences, University of the Punjab, Quaid-e-Azam campus, 54590 Lahore, Lahore Pakistan; 2 Psychiatry Department, Hawkes Bay DHB, Hastings, New Zealand; 3 Rainbow Obesity and Eating Disorders Centre, Lahore, Pakistan; 4 Department of Psychiatry, University of Minnesota, Minneapolis, MN USA; 5 Minneapolis Veterans Affairs Health Care System, Minneapolis, MN USA

**Keywords:** Schizophrenia, Bipolar disorder, Genetics, Pakistan, Consanguinity, Psychosis, Homozygosity

## Abstract

**Background:**

Psychiatric disorders are characterized by alteration in emotions, mood and behavior. Genetics is known to play a significant role in the development of psychiatric disorders. Genome-wide association studies have identified several loci associated with psychiatric illnesses. We hypothesize the existence of rare variants following Mendelian recessive mode of inheritance. These variants can be identified in families with multiple affected individuals born to unaffected consanguineous parents.

**Methods:**

We visited psychiatric outpatient departments of multiple hospitals in Lahore, Pakistan. We focused on psychosis, as it can occur in several DSM disorders such as schizophrenia, dementia and bipolar disorder. After clinical diagnosis by an American trained psychiatrist, detailed clinical assessments using Diagnostic Interview for Genetic Studies (DIGS), Diagnostic Interview for Psychosis and Affective Disorders (DI-PAD), Positive and Negative Syndrome Scale (PANSS), Hamilton Depression and Anxiety Rating Scale (HAM-D; HAM-A) were administered to all willing affected and unaffected participants.

**Results:**

We identified eight pedigrees with two or more psychotic individuals in each family. Clinical diagnoses determined by their psychiatrists included ten individuals with schizophrenia; four individuals with psychosis and bipolar disorder; and two patients with “unspecified psychosis.” The rating instruments rigorously confirmed the diagnosis of psychosis in the affected patients from the six families as well as the absence of psychotic disorders in unaffected individuals from the six families. We obtained DNA samples from willing members of all eight families for future genetic analyses.

**Conclusion:**

Our research highlights an alternative approach to discovery of rare recessively inherited genetic variants causing psychiatric disorders that have remained unidentified to date. These findings could illuminate underlying biological mechanisms leading toward development of targeted therapies in future.

## Introduction

Psychiatric disorders involve a combination of abnormal perceptions, thoughts, behaviors, emotions and their relationships [1]. According to the Diagnostic and Statistical Manual of Mental Disorders (DSM-5), there are almost 300 types of psychiatric illnesses. Two of these devastating disorders that frequently feature psychosis include schizophrenia (SCZ) and bipolar disorder (BD). The prevalence of schizophrenia in men and women is almost the same, whereas in individuals with bipolar disorder, females are slightly more commonly affected (52%) as compared to males (48%) [2]. Schizophrenia and bipolar disorder are estimated to affect between 20 million and 46 million people worldwide, respectively [2]. The prevalence of both illnesses is unknown in Pakistan as epidemiological data are not available. Since many patients do not seek treatment either due to fear of stigma, low income, or lack of access to health care facilities, therefore, many cases remain undiagnosed [3].

Schizophrenia is a chronic mental illness characterized by episodes of psychosis that often include hallucinations (auditory or visual); thought insertion or withdrawal; paranoia; disordered thinking; delusions; distorted perception of reality; catatonia; lack of emotional expression; and functional impairment [4]. Bipolar disorder consists of alternating depressive and manic episodes which are often separated by periods of normal mood. Depressive episodes can include insomnia; crying spells; poor eye contact; feelings of guilt, hopelessness, and worthlessness; negative rumination; and suicidal ideation [5]. Manic episodes are defined by severe hyperactivity; elevated mood; reduced need for sleep; grandiosity; high energy; and poor judgment, often with risk taking [6]. Psychotic symptoms include hallucinations, delusions, and thought disorder [7]. Clinically, symptoms must persist continuously for about six months for the diagnosis of schizophrenia. This six month time span can include a period of one month with only two or three typical symptoms (hallucinations, delusions, catatonia, flattening behavior, or social impairment) [8]. For a diagnosis of bipolar disorder, there should be at least two episodes affecting mood with at least one hypomanic/manic episode [9].

Multiple rating scales and diagnostic tools are used to assess psychopathology. These include the Diagnostic Interview for Genetic Studies (DIGS) with the modified Mini Mental Score Examination (mMMSE) [10]; Diagnostic Interview for Psychosis and Affective Disorders (DI-PAD); Positive and Negative Syndrome Scale (PANSS) [11], and Hamilton Depression and Anxiety Rating Scales (HAM-D; HAM-A) [12].

As determined by twin and family studies, genetics is a significant contributor to the development of schizophrenia [13] and bipolar disorder [14]. First degree relatives of affected individuals are more prone to develop schizophrenia (1–16%) [15] or bipolar disorder (8.34%) [16] as compared to those with no history of these disorders in their families.

Genome-Wide Association Studies (GWAS) have identified many common gene variants or copy number variants (CNVs) which are associated with psychiatric illnesses [17]. Exome sequencing of 265,218 patients suffering with 25 different psychiatric disorders and 784,643 controls revealed common risk variants, many of which were present in multiple affected individuals [18]. Genome-wide association studies have identified 145 susceptibility loci for schizophrenia [19, 20]. Genes associated with schizophrenia include *CHD7*, regulating neuronal differentiation [21]; *FURIN*, participating in neurodevelopment [22]; and *NMUR2* [23] and *SORCS3* [24], regulating receptors for sorting and signaling in neurons.

Genome-wide association studies have also identified major etiological factors for bipolar disorder. A study on genome-wide SNP data from 29,764 bipolar patients identified 30 significant loci associated with bipolar disorder [25]. Another meta-analysis of data from 41,917 patients identified 64 loci associated with bipolar disorder. The genes implicated in bipolar disorder included *CACNB2* and *KCNB1*, encoding proteins that regulate calcium ion-channels through modulation of calcium currents across neuronal membranes [26]; *HTR6*, encoding a serotonin receptor which mediates neurotransmission [27]; and *SLC1A2*, encoding a sodium dependent amino acid transporter with a role in the regulation and neurotransmission of glutamate [28]. These studies demonstrated that both schizophrenia and bipolar disorder are polygenic heritable disorders.

In Pakistan, a case control association study was performed on 508 unrelated schizophrenia patients and 300 healthy controls. Three SNPs, *rs4765905*, *rs6465084* and *rs1076560*, in *CACNA1C*, *DRD2* and *GRM3*, respectively, were associated with schizophrenia [29]. SNP genotyping was separately performed on a cohort of 120 sporadic bipolar patients and 120 controls in the Pakistani population. A SNP *rs1006737* in *CACNA1C* was associated with bipolar disorder [30].

In contrast to the association studies or heterozygous variants described to cause psychiatric disorders, only one locus has been mapped which segregates as a recessively inherited Mendelian disorder in three siblings diagnosed with schizophrenia [31]. Identification of recessively inherited pathogenic variants will help in determining phenotype-genotype relationship in psychiatric disorders. Around 60% of marriages in Pakistan are consanguineous out of which 80% are between first cousins [32]. Thus, this population is ideal to explore the contribution of rare autosomal recessive variants to psychiatric disorders [33]. By careful study of an individual family, the responsible ultra-rare variant in those who are homozygous has the potential to define the disease based on the target phenotype (e.g., psychosis), while heterozygous family members may show the associated phenotypic behavioral spectrum (e.g., anxiety). The present study is one of the first steps in this direction. There was no prior information about the feasibility in Pakistan of large scale recruitments, evaluations, and clinical characterization of multiplex, consanguineous families affected by psychiatric disorders.

We present clinical details of eight consanguineous families with apparent Mendelian inheritance of psychotic disorders. Previously, as a pilot study for proof of concept, we identified a consanguineous pedigree PSY01, with unaffected parents and two children with psychosis; five other siblings were unaffected. A rare, exonic, missense variant in *USP53* p.(Cys228Arg) predicted to be damaging and affecting an evolutionary conserved residue was identified that segregated as a Mendelian recessive trait (DOI: 10.22541/au.162626102.28489874/v1). This finding motivated the search for additional candidate gene variants associated with psychosis that are transmitted in a Mendelian recessive fashion in consanguineous families. As a preamble to future genetic studies, we comprehensively document feasibility, symptoms, and clinical features of other pedigrees recruited so far. We also note the barriers for recruitment and retention that were experienced as well as procedures deployed that may be useful in future work.

## Methods

### Family identification and clinical analysis

Ethical review and approval of the study was obtained from the committees at the respective universities which included the Institutional Review Board (IRB) at the School of Biological Sciences, University of the Punjab, Pakistan (IRB# 00005281, FWA 00010252) and University of Minnesota, Minneapolis, USA (FWA00000312). Outpatient departments and psychiatric wards at different hospitals of Lahore were visited. Several psychiatrists at the Punjab Institute of Mental Health (PIMH), Lahore, diagnosed the psychotic patients as having either schizophrenia or bipolar disorder. Later, detailed clinical assessments of six families were completed, whereas further clinical evaluations of participants from families PSYAK2 and PSYAK3 could not be carried out due to their refusal to undergo additional testing. Clinical assessments of families were performed using Diagnostic Interview for Genetic Studies (DIGS) [10]; modified MiniMental Status Examination (mMMSE) [34]; Diagnostic Interview for Psychosis and Affective Disorders (DI-PAD) [35]; Positive and Negative Syndrome Scale (PANSS) [11]; Hamilton Depression and Anxiety Rating Scales (HAM-D, HAM-A) ) [12]; and several additional rating tools used by investigators (SAS, AI).

A US trained Psychiatrist (SAS) took the lead in reviewing, conducting and training various structured clinical assessment tools to help establish the clinical diagnosis and to quantify the severity of psychiatric illnesses. He was assisted by a clinical psychologist (AI) and a graduate student (AK). They were fluent in both Urdu and English. The researchers (SAS, AI) interviewed the patients and relatives as part of the Diagnostic Interview for Genetic Studies (DIGS). Information was summarized in narrative form as well as in ratings from detailed assessment of the course of illness of the patients. Information from the medical records, from algorithmic diagnosis, and from interviews of relatives was used to score DSM-IV criteria as part of the Diagnostic Interview for Psychosis and Affective Disorders (DI-PAD). The Positive and Negative Syndrome Scale (PANSS) measured the severity of schizophrenia symptoms including positive symptoms, negative symptoms, and general psychopathology [11]. Positive symptoms included auditory or visual hallucinations, delusions (false beliefs), disorganized speech or behavior. Negative symptoms included affective flattening, anhedonia, social withdrawal, and inability for self-care [4]. Unaffected relatives were confirmed as cognitively intact with the modified MiniMental Status Examination (mMMSE; Folstein et al. 1975). The ratings and structured interviews were administered to the extent possible and as appropriate to the clinical setting. Descriptions of the patients’ symptoms are included to provide context; however, the diagnoses were based on strict criteria.

### Participants

Eight families, each with two affected individuals, participated in this study. In total, sixteen patients were diagnosed with the symptoms of psychosis; there were eight sets of unaffected parents. Nine participants were unaffected siblings of the patients. Inclusion criteria required per family a minimum of two individuals affected with psychosis born to unaffected consanguineous parents. Families having patients manifesting psychosis with accompanying diseases such as epilepsy were excluded. Family recruitment was performed with written informed consent of the participants or from the legally authorized representatives for minors or those patients who had severe ongoing symptoms. We visited all members at their homes for basic questioning about demographics and for obtaining blood samples. Written informed consent forms were signed by each participant.

Parents in each family were questioned about medical records, kinship, family history, children’s age on disease onset, and progression of symptoms in cases where patients were unable to provide a clear history because of illness severity. No probands were declared incompetent to provide consent. When competence was of potential concern, both probands and their parents consented. Blood samples from affected patients, their normal siblings, and unaffected parents were obtained in EDTA-treated BD vacutainer® (Becton, Dickinson and company, NJ, USA). DNA was extracted from blood using a standard sucrose lysis and salting out method [36]. These samples will be used for whole exome sequencing to find the causative genetic variants in the respective families. We will focus on rare variants (allele frequency less than 0.01, not homozygous in general population), predicted to be damaging by various software and segregating in a recessive fashion. The latter requires the variants to be homozygous in the affected individuals, heterozygous in the obligate carriers and wild-type or heterozygous in other unaffected individuals of the family—all confirmed by Sanger sequencing. The variants must not occur as a polymorphism in the local ethnically-matched population from where the samples were drawn.

For families PSYAK4, PSYAK5, PSYAK6, and PSYAK7 whole blood in red topped Becton Dickinson vacutainers was also obtained for extracting sera for future biochemical studies. Blood was placed undisturbed at room temperature for 30 min to clot. Samples were centrifuged at 2000 x g for 10 min using Novafuge BT337-5 K (Senova, Shanghai, China) at room temperature. The supernatant was immediately transferred to a clean polypropylene tube, aliquoted into 0.5ml tubes, and stored at -80 °C.

## Results

### Clinical manifestations of patients

We recruited members of eight families with at least two affected individuals suffering from psychosis either as a sole symptom complex, or as part of schizophrenia or bipolar disorder. Two families’ probands (i.e., two pairs) had psychosis with a history of affective symptoms consistent with bipolar disorder. Ten probands were diagnosed by their physicians as having schizophrenia. Altogether, sixteen affected individuals participated in this study with an equal number of males and females. Approximately 75% individuals had displayed aggressive behavior at various times, and 88% patients continued to manifest features of active psychosis. Parents in all cases were unaffected without lifetime history of a psychiatric disorder, as assessed by doctors at the Punjab Institute of Mental Health as well as through our clinical assessments (SAS & AI).

**Fig. 1:**
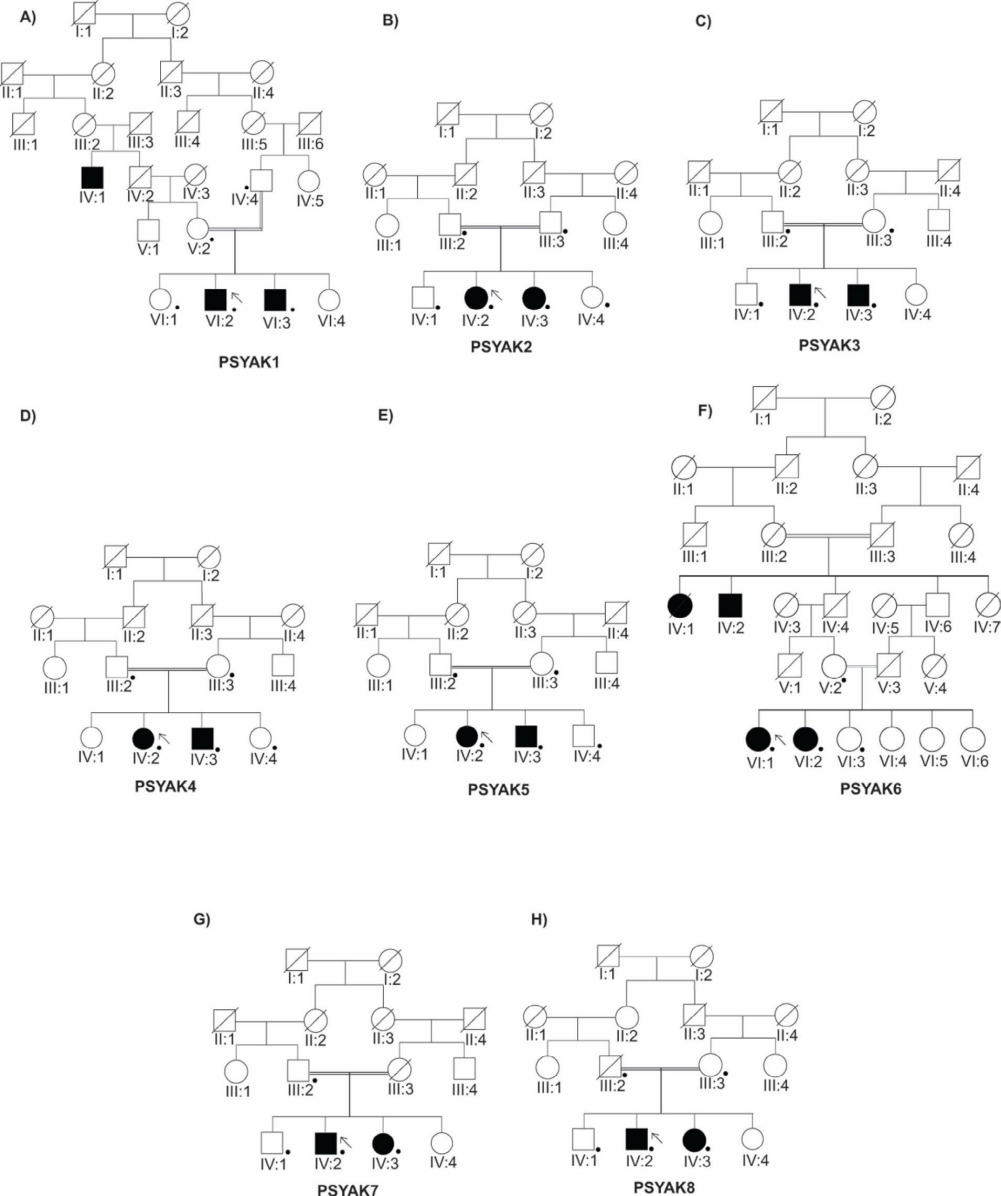
Pedigrees with multiple indivduals affected with psychiatric illnesses. Family trees of all eight participating pedigrees are shown. **(A)** Family PSYAK1: VI:2 and VI:3 are affected with psychosis. **(B)** Family PSYAK2: Individuals IV:2 and IV:3 are affected with schizophrenia. **(C)** Family PSYAK3: Two brothers are affected with schizophrenia. **(D)** Family PSYAK4: One sister IV:2 and one brother IV:3 are affected with paranoid schizophrenia. **(E)** Family PSYAK5: One sister IV:2 and one brother IV:3 are affected with bipolar disorder along with psychosis. **(F)** Family PSYAK6: Two sisters are affected with schizophrenia. **(G)** Family PSYAK7: Individuals IV:2 and IV:3 manifested schizophrenia not responsive to medication. **(H)** Family PSYAK8: Two individuals were affected with bipolar disorder with psychosis. Symbols (filled = affected, open = unaffected, cross = deceased, arrow = proband, half filled symbol = depression only, double line = consangineous marriage, and • = individual who participated in the study). In family PSYAK1, individual IV:1, and in family PSYAK6 individuals IV:1, IV:2 were reported to be affected by the family. These patients did not participate in the study and clinical assessment has not been performed for them

Family PSYAK1 (Fig. 1 A) had two affected individuals, VI:2 and VI:3, with symptoms of psychosis. Psychosis included auditory hallucinations (hearing voices from clouds and machines), delusions, and aggressive behavior such as beating children. The mother’s earliest recollection that something was wrong for individuals VI:2 and VI:3 was the onset of severe insomnia and aggressive behavior at the ages of 4 and 5 years, respectively. Gross psychosis was diagnosed by the doctors at the hospital at the ages of 11 and 17 years, respectively (Table 1). Individual VI:2 was unable to provide history because of poor insight and disruptive behavior. His mMMSE score was very low, because at the time of assessment, he was in a grossly psychotic state and was unable to respond to interview questions (he was hearing voices and was displaying aggressive behavior). The mMMSE is interpreted here to indicate the level of cooperation/performance at the time of interview not as a measure of cognitive function as used in non-psychotic elderly patients. His mother reported that the patient had a high-grade fever at the age of 4 years of uncertain significance to his psychiatric disorder. He was unable to attend school because of his cognitive and behavioral deficits. Patient VI:3 also exhibited with psychosis, intermittent angry outbursts, sadness, and irritability. The sadness occurred only for brief and intermittent periods of time and was less prominent than the ongoing severe psychosis. He was unable to care for himself, often begged for money from strangers, and beat other children. His mMMSE was 32 suggesting intact cognitive function.

**Table 1:** Clinical manifestations of the patients

Family	Patient	Sex	Age (Years)		Symptoms	Diagnosis	Current status	Medications
			**Current**	**Onset**				
PSYAK1	VI:2	M	19	11	Auditory hallucinations, aggressive behavior, agitation, insomnia	Psychosis	Stable on medicines	Lorazepam, Norfloxacin, Carbamazepine, Sodium Valproate
	VI:3	M	20	17		Psychosis	Stable on medicines	Lorazepam, Risperidone
PSYAK2	IV:2	F	25	12	Self-talking, auditory hallucinations, aggressive behavior, language dysfunction	Sz	Unstable	Fluoxetine, Procyclidine, Alprazolam, Sodium Valproate, Quetiapine
	IV:3	F	30	14		Sz	Unstable	Levodopa, Procyclidine, Alprazolam, Sodium Valproate
PSYAK3	IV:2	M	20	18	Self-talking, auditory hallucinations, aggressive behavior, self-smiling, unable to concentrate	Sz	Unstable	Carbamazepine, Fluvoxamine, Clonazepam, Levodopa, Procyclidine, Fluphenazine
	IV:3	M	28	26		Sz	Unstable	Levodopa, Procyclidine, Lorazepam
PSYAK4	IV:2	F	17	14	Self-talking, auditory hallucinations, delusions, self-smiling, catatonia	Sz	Unstable	Levodopa, Procyclidine, Sodium Valproate
	IV:3	M	20	19		Sz	Stable on medicines	Risperidone, Procyclidine
PSYAK5	IV:2	F	20	18	Mania, depression, unable to concentrate	BD	Stable on medicines	Levodopa, Clonazepam, Procyclidine
	IV:3	M	29	19		BD	Stable on medicines	Olanzapine, Procyclidine, Carbamazepine
PSYAK6	VI:1	F	25	24	Self-talking, auditory hallucinations, delusions, irrelevant talk, paranoia	Sz	Stable on medicnes	Olanzapine, Procyclidine, Sodium Valproate
	VI:2	F	26	23		Sz	Unstable	Levodopa, Procyclidine, Fluctuate, Carbamazepine, Fluvoxamine
PSYAK7	IV:2	M	36	24	Auditory hallucinations, aggressive behavior, paranoia, irrelevant talk	Sz	Unstable	Bromazepam, Nortriptyline
	IV:3	F	38	29		Sz	Unstable	Nortriptyline, Procyclidine
PSYAK8	IV:2	M	35	20	Irrelevant talk, Mania, Aggressive behavior, Abusive language	BD	Unstable	Lorazepam, Procyclidine
	IV:3	F	50	34		BD	Unstable	Procyclidine Lactulose, Haloperidol, Lorazepam, Mecobalamin, Propranolol

SZ, Schizophrenia, BD; Bipolar Disorder. Patients in families PSYAK1, PSYAK2, PSYAK3, PSYAK4 and PSYAK6 were prescribed antipsychotics (quetiapine, aripiprazole, or olanzapine). Some anti-psychotic drugs caused serious side effects including hormonal imbalances, heart disease cardiac arrhythmia and tardive dyskinesia. Therefore, the doctors switched sometimes the patients’ medication from antipsychotics to mood stabilizers or administered electroconvulsive therapy (ECT). In Pakistan, levodopa is used often to treat extrapyramidal side effects especially tremor

In family PSYAK2 (Fig. 1B), two females, IV:2 and IV:3, were affected with schizophrenia. Clinical manifestations included talking and smiling when alone, aggressive behaviors, delusions, auditory hallucinations, inability to focus, and formal thought disorder (Table 1). The family refused to engage in detailed clinical assessment because of beliefs associated with COVID-19. The family will be recontacted in the future, if they indicate their willingness for assessments.

Family PSYAK3 included two males with symptoms of schizophrenia (Fig. 1 C). Individual IV:2 had, auditory hallucinations (“someone is calling”), while his brother IV:3 had more negative symptoms including social withdrawal, lack of emotion, and flat affect. Both patients had aggressive behavior such beating others, using abusive language, smiling to themselves when alone. They were unable to concentrate for significant periods of time (Table 1). The family refused to take part in additional clinical assessment because of personal beliefs.

Family PSYAK4 (Fig. 1D) had two individuals, IV:2 and IV:3, with paranoid schizophrenia. Patient IV:2 had symptoms of self-talking; auditory and visual hallucinations (seeing her deceased sister and talking with her; imaginary interactions with others); paranoid delusions (life was in danger); self-smiling; social withdrawal; poor hygiene; intermittent fear; frequent suspiciousness; and at times catatonia. Her mMMSE was 26 suggesting moderate cognitive function. Individual IV:3 had symptoms of severe emotional dysregulation and disorganization; thought insertion and withdrawal; irrelevant or tangential speech; delusions (people gossip about him); poor insight; visual hallucinations (seeing his elder deceased sister moving around); social isolation; and persistently feeling intrusive attention of others (Table 1).

In family PSYAK5 (Fig. 1E), beside the cardinal symptom of psychosis, probands had significant affective symptoms that included mania and depression warranting the diagnosis of bipolar disorder. During the manic phase, the patients exhibited severe insomnia, aggression, circumstantial and tangential speech, high energy and hyperactivity, grandiosity, irritability, and abusive language. During the depressive phase, patients had feelings of sadness, somatic concerns, anxiety, emotional tension, lack of judgment and insight, tendency to frustration, catatonia, avoidance of social interactions, poor personal hygiene, and a general lack of interest. Both had discontinued their school studies because of mental health issues (Table 1). mMMSE scores of IV:2 and IV:3 were 26 and 24, respectively.

Family PSYAK6 (Fig. 1 F) had two females with symptoms of schizophrenia. These included echolalia, bizarre postures, incoherent speech, self-talking, auditory and visual hallucinations (imaginary individuals talking with each other), delusions (paranoia about patient’s murder; being followed; feeling someone being nearby); and loosening of associations. VI:1 was not able to take care of herself nor her children. Although individual VI:2 could attend to her hygiene, she was unable to take care of her children as well. VI:2 experienced multiple somatic delusions (Table 1) including a feeling that her hands were dead and that her body was unable to move. She often hallucinated peoples’ faces with white oval eyes. mMMSE scores for VI:1 and VI:2 were 13 and 02, respectively, which denotes significant cognitive impairment in both of these patients, but in the context of gross psychosis during assessment.

Family PSYAK7 (Fig. 1G) had two patients manifesting paranoid schizophrenia. One individual IV:2 showed symptoms of auditory hallucinations (hearing voices of clouds, voices of humans talking with each other), paranoia (other people are giving him poison to kill him), delusions (other people dislike him), aggression, disorganized thoughts, and illogical and sometimes incoherent speech. His sister IV:3 was very aggressive, abusive with negative symptoms, disorganized thoughts, incoherent speech, paranoia (someone is following her) and social withdrawal (Table 1). She was unable to answer questions of mMMSE because she was suffering from the primary psychotic episode with aggressiveness and severe paranoid features.

Family PSYAK8 (Fig. 1 H) had two individuals with history of psychosis and bipolar disorder. Both patients had euphoric symptoms during the manic phase along with loosening of association. They were overly aggressive, judgmental, and abusive. During depressive phases, the patients manifested loss of interest in activities and had frequent suicidal thoughts (Table 1). Individual IV:2 showed symptoms of emotional distress, functional deficits, agitation, abusiveness, anger/irritability, poor impulse control, defiant behavior, and grandiosity (“I am the ruler of the country; everyone should obey my order.”). He was hospitalized for four weeks and received psychiatric medications (Table 1). He had a mMMSE score of 03 which implicated poor insight and impaired cognitive function in a grossly psychotic episode. Individual IV:3 had six children. After the death of her child in 2002, she started having emotional disturbances and developed paranoid delusions about people (they were killing her children) that impaired daily functioning. She had symptoms of depression with psychotic features. The family reported that her issues started with low mood, sadness, crying episodes, poor hygiene, social isolation and delusions about family members (the belief that everyone is talking about her and wants to kill her children).

## Discussion

Psychosis is a cardinal symptom that can occur in several psychiatric disorders particularly schizophrenia and bipolar disorder with worldwide prevalence of 0.4–0.7 and 1%, respectively [37]. Several SNPs with small effect sizes and low penetrance have suggested polygenic inheritance for both schizophrenia [38] and bipolar disorder [26]. Multiple genes act in an additive manner in association with psychotic disorders (common disease-common allele hypothesis) [1]. Recently, another model has been proposed: the ‘‘Omnigenic Model.’’ According to this model, a few disease-causing variants act as core or nodes in gene regulatory networks, playing a central role in etiology of complex disorders. These exert direct effects on the phenotype of mental illnesses. Along with these core essential genes, peripheral genes are also present which are inter-connected and affect the function of the core genes [39]. Due to the peripheral genes affecting densely connected cellular mechanisms, the phenotype of core genes is altered [40]. This leads to a hypothesis: the “common disease-rare allele hypothesis,” i.e., the presence of core genes that are highly penetrant but present in small proportion of cases [1]. Thus, there could be some highly penetrant genetic variants which could directly affect the phenotype and play a role in the etiology of disease paving a way toward monogenic model of inheritance in a few cases. An example of a rare variant with high penetrance is a microdeletion on chromosome 22q11.2 which is associated with psychotic disorders. Its occurrence is low (1 in 4000 live births), but in 25% of affected cases, it is associated with schizophrenia or schizophrenia-like disorders [41].

A study examined a multiplex, consanguineous Saudi family with five siblings suffering from recessively inherited infantile parkinsonism-dystonia-2; mood instability; and developmental delay. Homozygous *SLC18A2* (*VMAT2*) variant p.(Pro387Leu) was found as the cause of the disorder in all five siblings. It is noteworthy that the heterozygous parents had major depression with no signs of the movement disorder observed in their children. Of interest, VMAT2 dysfunction had already been hypothesized as associated with depression [42, 43]. Also, a major hypothesis about the pathogenesis of mood disorders was the “Monoamine Theory of Depression” positing low synaptic monoamines might be causal to depression [44]. In this context, the variant appears pseudo-dominant for major depression [45]. Vesicular monoamine transporter 2 (VMAT2) is an integral part of the membranes of the synaptic vesicles and transports serotonin and dopamine into synaptic vesicles. This protein plays an essential role in mood stability and autonomic functions of body. The variant p.(Pro387Leu) was shown to impair the transport activity of VMAT2 [45]. This was one of the first rare single variants with large effect size associated with major depression.

In a large Scottish family with multiple affected individuals, a chromosomal translocation (1;11)(q42;q14.3) was found significantly associated with an increased risk of bipolar disorder, schizophrenia, and schizoaffective disorder [46]. After whole genome sequencing of 48 affected individuals from this family, the chromosomal translocation (1;11)(q42;q14.3) was discovered to disrupt three genes: *DISC1*, *DISC2* and *DISC1FP*. These gene disruptions were identified as highly penetrant with negative impacts on cognitive ability, neurodevelopment, and increased risk of psychiatric disorders [47].

Whole exome sequencing of 4264 schizophrenia patients and 1077 trios revealed three *de novo* loss of function mutations in *SETD1A*, (also known as *KMT2F*), which were absent in the general population. SETD1A plays a crucial role in methylation of histone H3 at Lys 4 (K4) [48]. Two pathogenic de novo variants in *ATP1A3* and missense variants in *FXYD* gene family (*FXYD1*, *FXYD2*) were also identified in 17 sporadic cases of childhood onset schizophrenia using whole exome sequencing. ATP1A3 regulates electrical excitability across neuronal membranes, and *FXYD* encodes a protein acting as an ion transport regulator [49]. Apart from variants on genes located on autosomes, those on X-chromosome have also been implicated in schizophrenia [50] .

Consanguinity is an important risk factor for schizophrenia because of the increased chances for homozygosity of deleterious alleles co-occurring in the affected individuals [51]. Similarly, consanguineous marriages increase the risk of bipolar disorder in the population [52], as well as of other psychiatric illnesses [53]. Gene mapping for autosomal recessive psychiatric disorder was performed for a Pakistani consanguineous family. This family presented multiple affected individuals with recessively inherited schizophrenia, hearing loss or epilepsy; either alone or in combination. Parents were without any lifetime diagnosis of a clinical disorder. The psychiatric disorder segregated independently of deafness as it mapped to chromosome 22q12.3-q13.3, while the deafness phenotype was mapped to chromosome 2p24 [31]. In a separate study on another Pakistani family, heterozygous missense variants *NRG3* p.(Glu651Lys) and *GRIN2A* p.(Arg1169Trp) were co-inherited in patients with schizophrenia from unaffected consanguineous parents who were carriers for only one of either variant [54]. Thus these results provide support for the hypothesis that families with psychotic disorders in Pakistan may ultimately help in identification of rare pathogenic variants inherited as recessive or digenic traits.

During our study, we observed guardians of many schizophrenic patients abandoned them at the hospital and did not keep further contact, even after repeated telephonic requests by the health personnel. We also faced challenges regarding recruitment of families with multiple individuals suffering with psychosis. Despite IRB approval for financial compensations, many people were reluctant to participate in the research. Some parents regarded their affected children as “mad” and impossible to treat. Other parents considered their children to be unaffected. They attributed the observed behavior to their child’s normal constitutional nature and therefore did not want to participate. A few individuals considered patients to have extraordinary abilities because of the patient’s ability to see deceased people and other imaginary objects. Among the participants that we did recruit, it was very difficult to clinically assess patients during the onset of symptoms. They were not able to answer many questions and parents had to be consulted. Frequently, multiple visits and rescheduling were required. It was challenging to obtain blood from some individuals due to the ongoing psychotic symptoms. We found taking smaller samples of blood (e.g., 5 ml) was better tolerated. Despite these barriers, we have successfully recruited eight families and were able to clinically assess six of them. These data also provide realistic expectations for determining power for future studies.

## Conclusion

Our research is designed to explore the genetics of the psychotic disorders segregating in consanguineous families with multiple affected individuals. The results of this research will establish whether these disorders are monogenic, omnigenic, or polygenic in our study families. Indeed, the approach appears tentatively successful in a family studied as a pilot to this work. If few or no rare homozygous likely pathogenic variants are found segregating with the disorders in these consanguineous pedigrees, this information will be important regardless. If rare pathogenic variants are identified, the data will provide candidate genes for association with phenotype in larger cohorts as well. Ultimately, identification of disease gene variants will open up diagnostic and therapeutic opportunities for psychiatric disorders.

## Data Availability

Not Applicable.
